# *Hierarchical Meta-Storms* enables comprehensive and rapid comparison of microbiome functional profiles on a large scale using hierarchical dissimilarity metrics and parallel computing

**DOI:** 10.1093/bioadv/vbab003

**Published:** 2021-05-12

**Authors:** Yufeng Zhang, Gongchao Jing, Yuzhu Chen, Jinhua Li, Xiaoquan Su

**Affiliations:** 1 College of Computer Science and Technology, Qingdao University, Qingdao, Shandong 266071, China; 2 Single-Cell Center, Qingdao Institute of BioEnergy and Bioprocess Technology, Chinese Academy of Sciences, Qingdao, Shandong 266101, China

## Abstract

Functional beta-diversity analysis on numerous microbiomes interprets the linkages between metabolic functions and their meta-data. To evaluate the microbiome beta-diversity, widely used distance metrices only count overlapped gene families but omit their inherent relationships, resulting in erroneous distances due to the sparsity of high-dimensional function profiles. Here we propose *Hierarchical Meta-Storms* (HMS) to tackle such problem. HMS contains two core components: (i) a dissimilarity algorithm that comprehensively measures functional distances among microbiomes using multi-level metabolic hierarchy and (ii) a fast Principal Co-ordinates Analysis (PCoA) implementation that deduces the beta-diversity pattern optimized by parallel computing. Results showed HMS can detect the variations of microbial functions in upper-level metabolic pathways, however, always missed by other methods. In addition, HMS accomplished the pairwise distance matrix and PCoA for 20 000 microbiomes in 3.9 h on a single computing node, which was 23 times faster and 80% less RAM consumption compared to existing methods, enabling the in-depth data mining among microbiomes on a high resolution. HMS takes microbiome functional profiles as input, produces their pairwise distance matrix and PCoA coordinates.

**Availability and implementation:**

It is coded in C/C++ with parallel computing and released in two alternative forms: a standalone software (https://github.com/qdu-bioinfo/hierarchical-meta-storms) and an equivalent R package (https://github.com/qdu-bioinfo/hrms).

**Supplementary information:**

Supplementary data are available at *Bioinformatics Advances* online.

## 1 Introduction

Microbiome functional profiling is thought to be superior to taxonomic profiling (Langille, 2018), for it quantifies the genes and metabolic pathways of microorganisms that answers ‘what a microbial community can do’ (Knight *et al.*, 2018), linking the dynamics of metabolic activities to environment conditions (Fuhrman, 2009) and health status (Lloyd-Price *et al.*, 2016). Functional features can be directly parsed out from metagenomic shotgun whole-genome sequencing (WGS) data by tools like HUMAnN (Abubucker *et al.*, 2012; Franzosa *et al.*, 2018), yet limited by the high experiment and computation cost (Morgan and Huttenhower, 2012). Amplicon-based methods [e.g. PICRUSt (Douglas *et al.*, 2020; Langille *et al.*, 2013), Taxa4Fun (Asshauer *et al.*, 2015), PanFP (Jun *et al.*, 2015)] can infer molecular functions from 16S rRNA gene, however, the accuracy is deviated from WGS approaches due to amplification bias and inadequate amplicon-genome linkages. Recently, Meta-Apo (Jing *et al.*, 2021) was developed for the calibration of amplicon-derived functions, which provides a new solution for large-scale functional survey with cheap cost of amplicon sequencing and high resolution of WGS, thus enables the understanding of the global microbiome data space on a broader range (Su *et al.*, 2020).

**Fig. 1.**
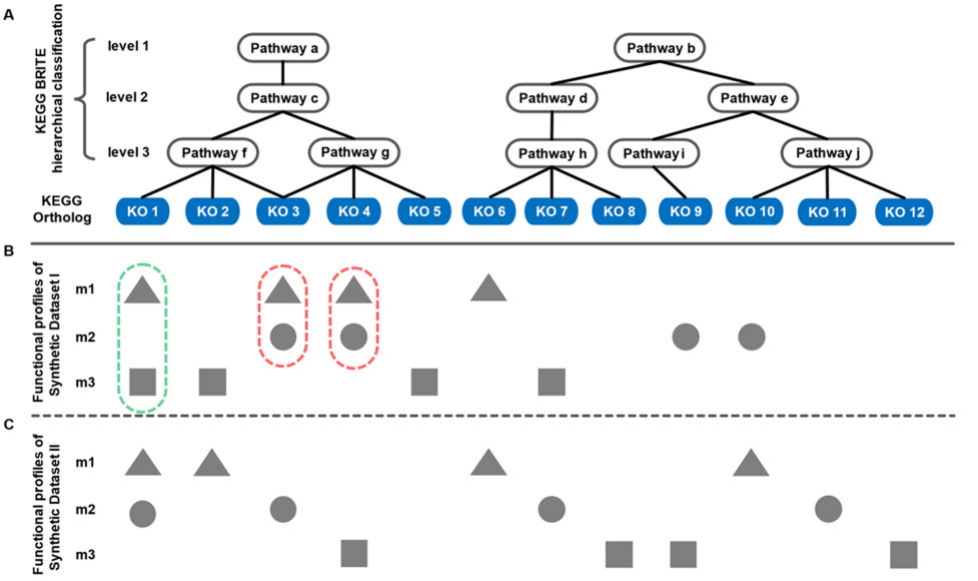
The hierarchical structure of functional profiles. **(A)** KOs and KEGG BRITE 3-level classification of pathways. **(B)** For Synthetic Dataset I, group m1 shares more KOs with m2 than m3, but m1 is more similar to m3 since their KOs belongs to the exactly the same metabolic pathway branches. **(C)** For Synthetic Dataset II, it is spares and zero-inflated for KO distribution that few KOs are in common among different groups

Functional beta-diversity analysis on massive number of microbiomes interprets the relations between metabolic features and their meta-data (Huttenhower *et al.*, 2012). How to quantitatively assess functional dissimilarities (or distances) among microbiomes is the basis for beta-diversity analysis. Commonly used geometry- or statistics-based metrics such as Jensen–Shannon Divergency (JSD) and Bray–Curtis distance mainly rely on detecting the overlapped gene families (e.g. KEGG Ortholog; KO) but ignore their inherent connections or relationships, causing the erroneous results in beta-diversity pattern. Specifically, as the distribution of global microbes is sparse among ecosystem (Thompson *et al.*, 2017), it is natural that two microbiomes may share few identical KOs due to their distinct community members (Hacquard *et al.*, 2015). However, we cannot simply assert a small similarity between them, since different gene families may also contribute to the same metabolic pathway (Fig. 1A). On the other side, previously we have introduced phylogeny-based distance algorithms [e.g. Meta-Storms (Su *et al.*, 2012) and Dynamic Meta-Storms (Jing *et al.*, 2020)] for taxonomical comparison using evolutionary affinity of microbes, but such a definite tree-like structure of species is not applicable for functional profiles, for a single gene family is always involved in multiple metabolic pathways (Fig. 1A).

After calculating a pairwise distances, multi-dimensional scaling methods like Principal Co-ordinates Analysis (PCoA) are always employed to illustrate and visualize the beta-diversity pattern of microbiomes and their phenotypes such as environmental condition or host healthy status. PCoA maps all samples into a 2- or 3-dimensional coordinate system by dimension reduction of pairwise distance matrix. Nevertheless, most existing PCoA implementations [e.g. ‘ape’ package (Paradis *et al.*, 2004) and ‘vegan’ package (Dixon, 2003) in R] have not been adapted to multi-core processors that widely exists in current computer systems, causing an low utilization rate of advanced hardware.

## 2 Methods

In this work, we propose Hierarchical Meta-Storms (HMS) software for rapid and comprehensive beta-diversity analysis on microbiome functional profiles. This software contains two core components, (i) a hierarchical dissimilarity algorithm that comprehensively calculates functional distances among microbiomes by employing a multi-level metabolic pathway hierarchy and (ii) a fast PCoA implementation optimized by multi-thread parallel computing for thousands of samples. HMS takes microbiome functional profiles [e.g. parsed by HUMANn2 (Franzosa *et al.*, 2018), PICRUSt2 (Douglas *et al.*, 2020) or Meta-Apo (Jing *et al.*, 2021)] as input, produces their pairwise distance matrix and PCoA coordinates.

### 2.1 Hierarchical-based algorithm for comprehensive dissimilarity calculation of microbiome functional profiles

After functional profiling, a microbial community is represented by a series of functions or gene families (e.g. KO) and their relative abundances. Each function contributes to multiple metabolic pathways, which are pre-defined and annotated by a hierarchical structure (e.g. KEGG BRITE 3-level hierarchical classification that integrated in this package; Fig. 1A). When comparing two microbial communities, HMS firstly measures their difference by the relative abundances of gene families (e.g. KO in Fig. 1A) using Bray–Curtis distance (*Dist_0_* in Equation 1; Supplementary Equation S1). To consider the effect of inter-function relations on microbiome distances, gene families are then collapsed to pathways for further dissimilarity calculation and iterated over all levels in the hierarchical structure. Specifically, for each KO, HMS adds its relative abundance to bottom-level (level 3) pathways linked with this KO and obtains the Bray–Curtis distance of this pathway level (*Dist_i_* in Equation 1, *i*** **=** **3) after adding all KOs. This procedure is then iterated on higher layers until the top level (level 1), respectively, and the overall dissimilarity between two samples is the weighted mean value of distances on gene family and all pathway levels (Equation 1).

(1) $$Dist=\;\frac{\;\sum_{i=0}^{3}{\mathrm{Dist}}_{i}\;\times {\;W}_{i}}{\sum_{i=0}^{3}W_{i}}$$

In this equation, since relative abundances of upper-level pathways are the linear combination of lower levels that reduced the functional resolution, we set a linear weight on the three-level BRITE pathways according to their level (e.g. *W_i_ = i, i*** **>** **0), and set the weight of KO as 4 (*W_0_* = 4) for its highest resolution (refer to Supplementary Methods for detailed time complexity analysis).

### 2.2 Parallel computing strategy for pairwise comparison and beta-diversity pattern parsing

A pairwise distance matrix contains the HMS dissimilarities among all sample pairs, which is fundamental for beta-diversity analysis. In an *n*-dimension pairwise distance matrix (denoted by *DistMatrix* in Equation 2) for *n* samples, each element is a dissimilarity value between two microbiomes, e.g. *d_ij_* denotes the distance between sample *i* and sample *j* (1 <*i*** **<** ***n* and 1 <*j*** **<** ***n*). The distance matrix is symmetric (*d_ij_* = *d_ji_*) and the diagonal elements are always zeros (*d_ii_* = 0), so only half of the matrix (e.g. the upper triangle matrix with $\frac{n*(n-1)}{2}$ elements) need to be generated.

(2) $$\mathrm{DistMatrix}=\;\left[\begin{array}{ccc} d_{11} & \cdots & d_{1n}\\ \vdots & \ddots & \vdots \\ d_{n1} & \cdots & d_{nn} \end{array}\right]$$

Then based on the pairwise distance matrix, the PCoA maps all microbiome samples to a lower *k*-dimension coordinate system (e.g. *k*** **=** **2 or 3 space; refer to Supplementary Methods for detailed procedure) to visualize and interpret their relations according to meta-data, e.g. whether samples could be sorted by environmental conditions or healthy status.

As the operation of each element in a distance matrix and PCoA dimension reduction is independent and irrelevant to others, the whole calculation procedure can be divided into sub-tasks and parallelized for speedup. In our implementation, HMS assigns each of the sub-tasks to one thread and invocates multiple threads by POSIX OpenMP library on multi-core CPUs for parallel computing. Furthermore, all computing sub-tasks are dynamically scheduled at the running time (by setting OpenMP scheduling as ‘dynamic’) for a balanced loading of CPU cores to ensure a high efficiency.

## 3 Results

### 3.1 Datasets and experiment design

In this work, we prepared two synthetic datasets and three real datasets (Table 1) to assess the performance of HMS in accuracy, comprehensiveness, running time and memory usage for functional beta-diversity analysis. Synthetic Dataset I contains functional KO profiles of 30 artificial microbiomes that evenly divided into three groups (m1, m2 and m3). KO compositions of each group followed the pattern as Figure 1B and samples in the same group consist of similar KOs but only with subtle variations on relative abundances. Synthetic Dataset II was simulated in the same way as Dataset I by following the community pattern in Figure 1C. Real Dataset I contains KO profiles of 20 000 microbiomes randomly selected from Microbiome Search Engine database (mse.ac.cn) (Su *et al.*, 2018). Real Datasets II and III were produced by Human Microbiome Project Phase 1 (Turnbaugh *et al.*, 2007): Read Dataset II contains KO profiles of 5350 human microbiomes (gut, oral, skin and vagina) inferred from 16S rRNA gene amplicons by PICRUSt2 (Douglas *et al.*, 2020), and Real Dataset III contains KO profiles of 2354 human microbiomes (gut, oral, skin and vagina) reconstructed from WGS data by HUMANn2 (Franzosa *et al.*, 2018). Since R has already been widely applied in bioinformatics analysis(Kramer *et al.*, 2014), we set R-based distance methods (Bray–Curtis, Cosine, Euclidean and JSD) and PCoA (‘vegan’ package and ‘ape’ package in R) as benchmarks for comparison to HMS.

**Table 1.** The datasets to assess the performance of HMS on accuracy, running time and memory usage

Dataset	No. of samples	Sample source	Sample type
Synthetic Dataset I	30	Synthetized from KO gene families	Synthetic sample
Synthetic Dataset II	30	Synthetized from KO gene families	Synthetic sample
Real Dataset I	20 000	Microbiome Search Engine database	Real sample, inferred from 16S rRNA gene amplicons by PICRUSt2
		(mse.ac.cn)	
Real Dataset II	5350	Human Microbiome Project, Phase I	Real sample, inferred from 16S rRNA gene amplicons by PICRUSt2
Real Dataset III	2354	Human Microbiome Project, Phase I	Real sample, reconstructed from WGS data by HUMANn2

### 3.2 Benchmark the accuracy and comprehensiveness of hierarchical-based dissimilarity using synthetic data

#### 3.2.1 Erroneous pattern among groups

**Fig. 2.**
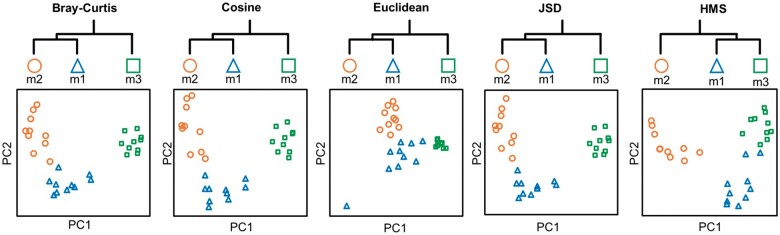
PCoA and hierarchical clustering results on Synthetic Dataset I. Only HMS generates the expected beta-diversity pattern that dist(m1, m2) > dist(m1, m3)

For Synthetic Dataset I, as shown in Figure 1B, samples in group m1 share more common KOs with group m2 (2 KOs, green-dotted circle) than m3 (only 1 KO, red-dotted circle). But actually, the overall metabolic functions and pathways of group m1 are more similar to those of m3 since their gene families belong to the identical pathways on KEGG BRITE hierarchy branches, e.g. on level-3 all pathways are exactly the same; in contrast, m1 and m2 differed from each other for only shared 2 of 5 pathways on level-3. Here we calculated pairwise distances of all 30 samples in Synthetic Dataset I using four metrics of Bray–Curtis, Cosine, Euclidean, JSD and our HMS, respectively. From the PCoA and clustering (‘hclust’ function in R) results in Figure 2, we observed that only HMS correctly generated the expected relations among the three groups that dist(m1, m2) > dist(m1, m3), but Bray–Curtis distance, Cosine distance, Euclidean distance and JSD delivered the opposite results thus lead to erroneous patterns.

#### 3.2.2 Anomalous layout by sparse distribution

**Fig. 3.**
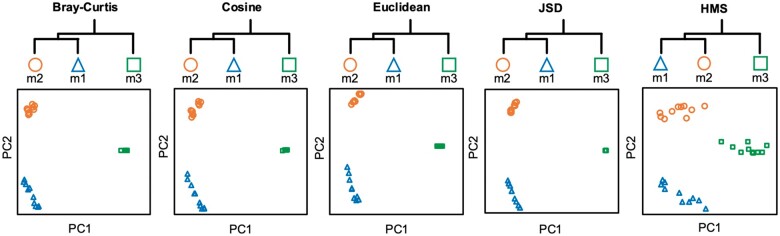
PCoA and hierarchical clustering results on Synthetic Dataset II. Layouts of the benchmark methods are anomalous that failed in assessing the beta-diversity within each group, while HMS kept the in-group variations

For Synthetic Dataset II, KO distribution of samples was spares and zero-inflated (Xu *et al.*, 2015) that few KOs were in common among different groups (Fig. 3). As the dataset design, group m1 was close to m2 for their KOs were located at the same hierarchical branches than m3, and all methods produced the expected relation of dist(m1, m2) < dist(m1, m3) among three synthetic groups. However, PCoA layouts of the benchmark methods were anomalous that failed in assessing the beta-diversity within each group. For example, 10 samples of group m3 were clustered as fully overlapped points in benchmark PCoA coordinates. The reason was that the high dimensionality and sparsity of KO profiles enlarged distances among groups, while omitted the variation of samples in the same group. By taking additional upper-level metabolic pathways for distance measurement, HMS differentiated three groups while in-group beta-diversity was kept, thus reduced the zero-inflation effects of functional profiles that may disturb the beta-diversity pattern.

### 3.3 Benchmark the efficiency of parallel computing in distance matrix calculation and PCoA

**Fig. 4.**
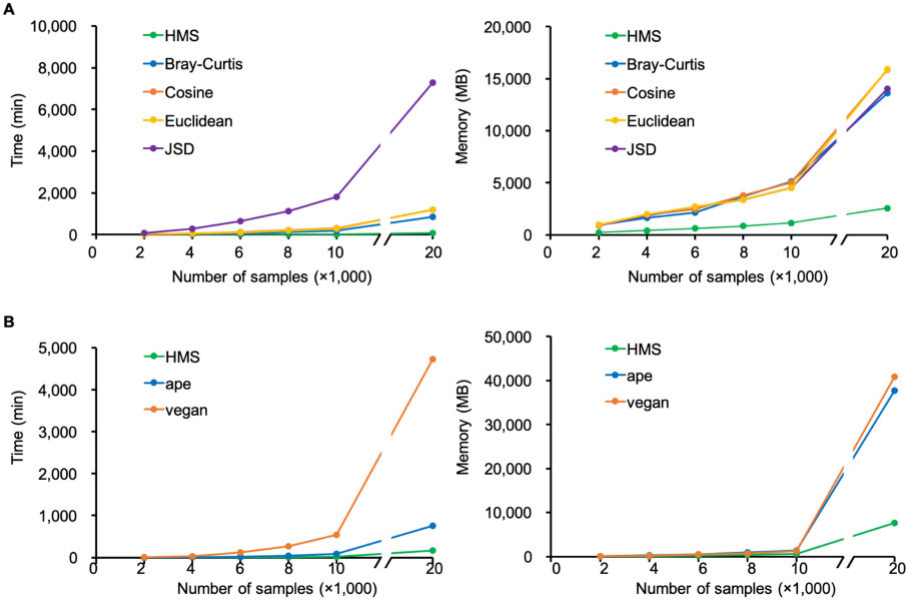
Running time and peak memory usage of distance matrix calculation and PCoA. **(A)** For pairwise comparison, HMS is 36 times faster than the benchmark methods, yet saves over 82% memory usage. **(B)** For PCoA, HMS is 17 times faster than the benchmark methods and saves over 80% memory usage

To test the efficiency of the HMS software, we performed the distance matrix calculation and PCoA for different numbers of samples (from 2000 to 20 000) that randomly selected from Real Dataset I, and compared the total running time and maximum RAM usage to the benchmark methods of R-based distance and PCoA methods. All tests were performed on a single non-shared computing node with 80 threads (supported by 40 physical cores). Calculations on each sample number were repeated for 10 times, and the mean running time and memory consumption was obtained to avoid interferences from computer system. When processing 20 000 samples, HMS completed the pairwise distance matrix in 73 min that is 36 times faster than the benchmark methods, yet saved over 82% memory by a peak RAM usage of 2.5 GB (Fig. 4A; Supplementary Fig. S1A in log-scale; Supplementary Table S1). Then for the PCoA, HMS also exhibited the advantages in both speed (161 min, 17 times faster) and resource usage (7.5 GB, 80% less memory) compared to the benchmark methods (Fig. 4B; Supplementary Fig. S1B in log-scale; Supplementary Table S2). Therefore, HMS achieved an overall 23 times speedup (HMS: 3.89 h; mean of benchmark methods: 89.55 h) in parsing the functional beta-diversity pattern, which is crucial and valuable as the number of metagenomic functional profiles is exponentially growing.

### 3.4 Performance of functional beta-diversity analysis on real datasets

**Fig. 5.**
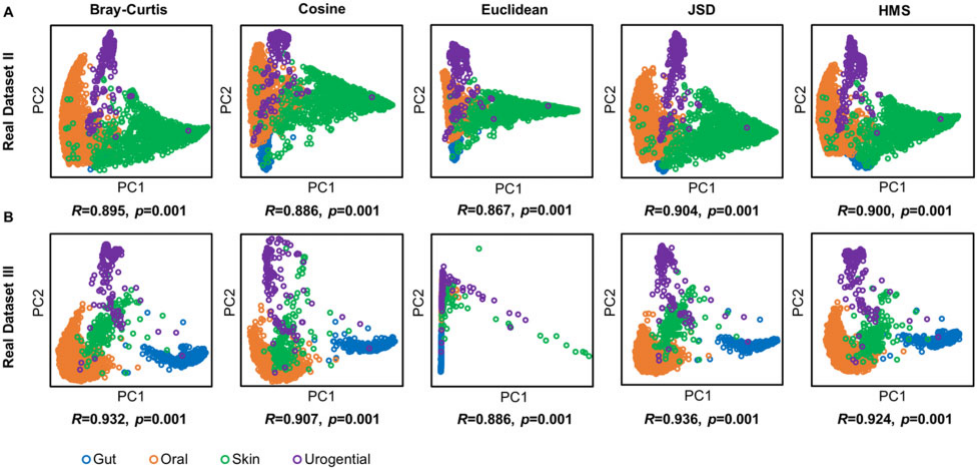
PCoA of real human microbiome functional profiles analysed from (**A**) 16S rRNA gene amplicons in Real Dataset II and (**B**) WGS in Real Dataset III

**Fig. 6.**
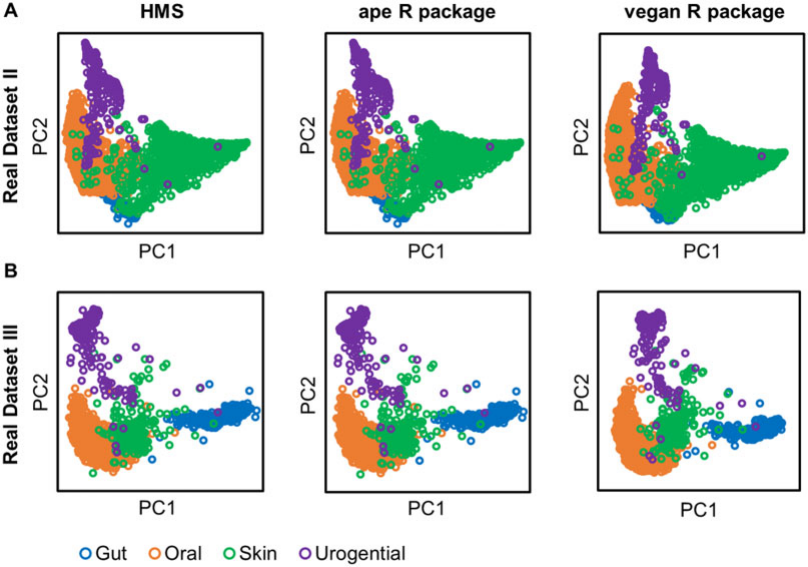
PCoA of HMS and benchmark methods are strongly correlated

Furthermore, the capability and reliability of HMS in processing real microbiomes were verified by Real Datasets II and III in two subtests. *Subtest 1. PCoA of different distance matrices:* For the two real datasets, we calculated their pairwise distance matrix by HMS and aforementioned three benchmark distance metrics, and plotted the principle coordinates by a unified PCoA method (‘vegan’ package in R). For both two real datasets, the hierarchical dissimilarity algorithm was able to cluster and distinguish microbiomes by their source habitats (ANOSIM test *R*** **=** **0.90 and *R*** **=** **0.92 for Real Datasets II and III, *P*-value < 0.01; ‘*anosim*’ function of ‘vegan’ package in R), as well as Bray–Curtis, Cosine and JSD (Fig. 5), showing the applicability of HMS distance on real microbiomes. Notably, the Euclidean distance produced a disordered PCoA layout on Real Dataset III (WGS data of human microbiome) due to the sparse KO profiles among different body sites (Thompson *et al.*, 2017). *Subtest 2. Different PCoA methods on the same distance matrix:* We then took the HMS distance matrix as input, and deduced the principle coordinates by PCoA of HMS, ‘vegan’ package and ‘ape’ package in R, respectively, and assessed the consistency of three results using Monte-Carlo test (10 000 times permutation; ‘*procuste.randtest*’ function of ‘ade4’ package in R). Results in Figure 6 suggested that results by HMS and benchmark methods were strongly correlated without significant difference (*R* > 0.99, *P*-value < 0.01), hence the HMS PCoA provides the interpretation of beta-diversity pattern with equivalent precision as other implementations but much higher speed (Fig. 4B).

## 4 Conclusion and discussion

A massive number of microbiomes from various habitats have already been generated to characterize the dynamics between microbial metabolic features and their surroundings. Typically, microbiome functional profiles are sparse since gene families are unique and non-shared across samples from multiple habitats. Although some approaches like Carnelian (Nazeen *et al.*, 2020) can find a few metabolic pathways as biomarkers for different samples, such a small fraction is not adequate for the ‘whole-community-level’ comparison when using geometry- or statistics-based metrics (Xu *et al.*, 2015). In this work, we proposed a hierarchical-based algorithm for comprehensive distance measurement among microbiome functional features, which provides higher sensitivity in detecting variations in upper-level metabolic pathways but ignored by other metrics, reducing the zero-inflation effects of functional profiles. This release version has integrated the KOs and BRITE pathways, making HMS direct accepts functional profiles from HUMANn2 (Franzosa *et al.*, 2018), PICRUSt2 (Douglas *et al.*, 2020; Langille *et al.*, 2013) or Meta-Apo (Jing *et al.*, 2021) as input. The COG (Cluster of Orthologous Genes) (Galperin *et al.*, 2015) and MetaCyc (Caspi *et al.*, 2016) pathways will also be supported in the further versions, as well as the customized hierarchical functional annotations to expand the usability and compatibility.

On the other hand, it is possible that a single study can survey over 10 000 microbiomes, e.g. Earth Microbiome Project (Thompson *et al.*, 2017) and American Gut Project (McDonald *et al.*, 2018). Such high throughput of multi-habitat profiling also introduces new challenges for computing the similarity of microbial functions in speed. Although the hierarchical-based algorithm is theoretically more complex and time-consuming (refer to ‘running time of HMS with single-core’ in Supplementary Tables S1 and S2), the optimized parallelization strategy achieved a 23× faster compared to the existing R-based implementations that only allow serial runs. Notably, the parallelized PCoA module in HMS could also be used as a general-purpose multi-dimensional scaling method for beta-diversity illustration and visualization. Therefore, by two alternative implementations of standalone software and R plug-in package, the HMS enables the beta-diversity pattern depiction for thousands of microbiomes on a single computing node or even a personal computer, which promotes the understanding of roles and effects of microbial communities from functional aspect on a large scale.

## Code and data availability

The HMS software is available at GitHub repository under a GNU GPL license. It is released in two alternative forms: a standalone software package and an equivalent R package for invocation in R scripts.

The standalone package (https://github.com/qdu-bioinfo/hierarchical-meta-storms) is developed by C++ for direct installation and use under Linux or MAC operating systems. A shell-based automatic installer is integrated in the package for easy installation by only one-line command. In the current version, the complete bacteria KOs and BRITE hierarchical annotations of pathways have been integrated. After installation, the HMS takes microbiome functional profiles of KO relative abundance as input, computes and outputs a pairwise distance matrix and the principle coordinates of PCoA for all input samples. The detailed tutorial is available in the package as well as an example demo dataset for quick start.

In addition, we also encapsulate the C++ source codes as an R package (https://github.com/qdu-bioinfo/hrms) by RcppArmadillo framework, making the kernel functions of distance calculation and PCoA callable by R interpreter in both R terminal and R scripts. Coupled with various R-based plug-ins of statistics, machine learning and graph plotting, the HMS will contribute in further developments and applications of microbiome functional data mining.

All datasets in this manuscript are also publicly available at GitHub (https://github.com/qdu-bioinfo/hierarchical-meta-storms).

## Supplementary Material

vbab003_Supplementary_Data
