# Supplementary material for: Hierarchical Meta-Storms enables comprehensive and rapid comparison of microbiome functional profiles on a large scale using hierarchical dissimilarity metrics and parallel computing
Source: Bioinform Adv. 2021 May 12;1(1):vbab003. doi: 10.1093/bioadv/vbab003 (PMC9710644; doi:10.1093/bioadv/vbab003)
Supplement: vbab003_Supplementary_Data [file vbab003_supplementary_data.docx]

**Supplementary Materials for**

***Hierarchical Meta-Storms* enables comprehensive and rapid comparison of** **microbiome functional profiles on a large scale using** **hierarchical dissimilarity metrics and parallel computing**

Yufeng Zhang^1^, Gongchao Jing^2^, Yuzhu Chen^1^, Jinhua Li^1 *^, Xiaoquan Su^1, 2 *^

^1^ College of Computer Science and Technology, Qingdao University, Qingdao, Shandong, China, 266071

^2^ Single-Cell Center, Qingdao Institute of BioEnergy and Bioprocess Technology, Chinese Academy of Sciences, Qingdao, Shandong, China, 266101

^*^Correspondence to Xiaoquan Su (suxq@qdu.edu.cn) and Jinhua Li (lijh@qdu.edu.cn)

**Supplementary methods**

*Bray-Curtis dissimilarity*

The Bray-Curtis algorithm, previously introduced by J.R. Bray and J. T. Curtis, is a procedure that computes the similarity of two microbiome samples (e.g *A* and *B*) based on the overlap of the relative abundances of functions (*equation S1*).

| ${Dist}_{Bray-Curtis}(A, B)=1-2 \times\frac{\sum min(S_{A,i},S_{B,i})}{\sum S_{A,i}+\sum S_{B,i}}$ | *(S1)* |
| --- | --- |

in which *S_A,i_* and *S_B,i_* mean the relative abundance of the *i*-th function from sample *A* and *B*.

*Time complexity of hierarchical-based dissimilarity*

The ordinary distance metrics of Bray-Curtis, Cosine or JSD only take end-level functions (e.g. KO), thus the time complexity of calculating the distance of a single sample pair is O(*N*), in which *N* is the number of functions. The HMS dissimilarity considers additional information of the multi-level metabolic pathway hierarchy among functions. On a pathway level (e.g. level 3 in **Fig. 1A**), we suppose that each feature was contributed from *m* features of its previous level. For example, all *N* KOs were combined into *N*/*m* level-3 pathways, and then combined into *N*/*m^2^* level-2 pathways. Notably, the computing time on one level is 2 * *N*, that is one *N* for distance measurement, and another *N* for feature combination to the higher level. Therefore, the expected complexity of HMS algorithm could be estimated as O(*N*) by *equation S2*.

| $T\left( N \right)=2\times N+2\times\frac{N}{m} +2\times\frac{N}{m^{2}}+2\times\frac{N}{m^{3}}+\ldots$  $=2N \times\sum_{i=0}^{\left\lceil\log_{m} N \right\rceil} \frac{1}{m^{i}}$  = $2N\times\frac{1-\left( \frac{1}{m} \right)^{\left\lceil\log_{m} N \right\rceil}}{1- \frac{1}{m}} = 2N\times\frac{1-\frac{1}{N}}{1- \frac{1}{m}}$  $= 2N\times\frac{m\left( N-1 \right)}{N\left( m-1 \right)} =\frac{2m}{m-1} \times\left( N-1 \right)$  $\approx O(N)$ | *(S2)* |
| --- | --- |

*Implementation of PCoA procedure*

In PCoA, firstly we make *DistMatrix’* (*equation S3*) consisted of the squares of elements in the original *DistMatrix*. After that, we solved the eigenvalues *Λ* (*equation S4*) and eigenvectors *U* (*equation S5*) of *DistMatrix’* by C++ Eigen library (<http://eigen.tuxfamily.org>). Picking the top *k* largest elements in eigenvalues *Λ* and their corresponding columns of eigenvectors, the *k*-dimension principle coordinates could be produced by *equation S6*, in which the *i*-th row is the coordinates of *i*-th sample in the *k*-dimension space.

| $Di{stMatrix}^{'}=\left[ \begin{matrix} d_{11}^{2} & \cdots& d_{1n}^{2} \\ \vdots& \ddots& \vdots\\ d_{n1}^{2} & \cdots& d_{nn}^{2} \end{matrix} \right]$ | *(S3)* |
| --- | --- |
|  |  |
| $\Lambda=[\lambda_{1},\lambda_{2},\ldots\ldots,\lambda_{n}]$ | *(S4)* |
|  |  |
| $U= \left[ U_{1}, U_{2}, \ldots, U_{n} \right] = \left[ \begin{matrix} u_{11} & \cdots& u_{n1} \\ \vdots& \ddots& \vdots\\ u_{1n} & \cdots& u_{nn} \end{matrix} \right]$ | *(S5)* |
|  |  |
| $PC=\left[ U_{i}\times\sqrt{\lambda_{i}} \right]=\left[ \begin{matrix} u_{11}\sqrt{\lambda_{1}} & \cdots& u_{k1}\sqrt{\lambda_{k}} \\ \vdots& \ddots& \vdots\\ u_{1n}\sqrt{\lambda_{1}} & \cdots& u_{kn}\sqrt{\lambda_{k}} \end{matrix} \right]$ | *(S6)* |
|  |  |

**Supplementary figures**


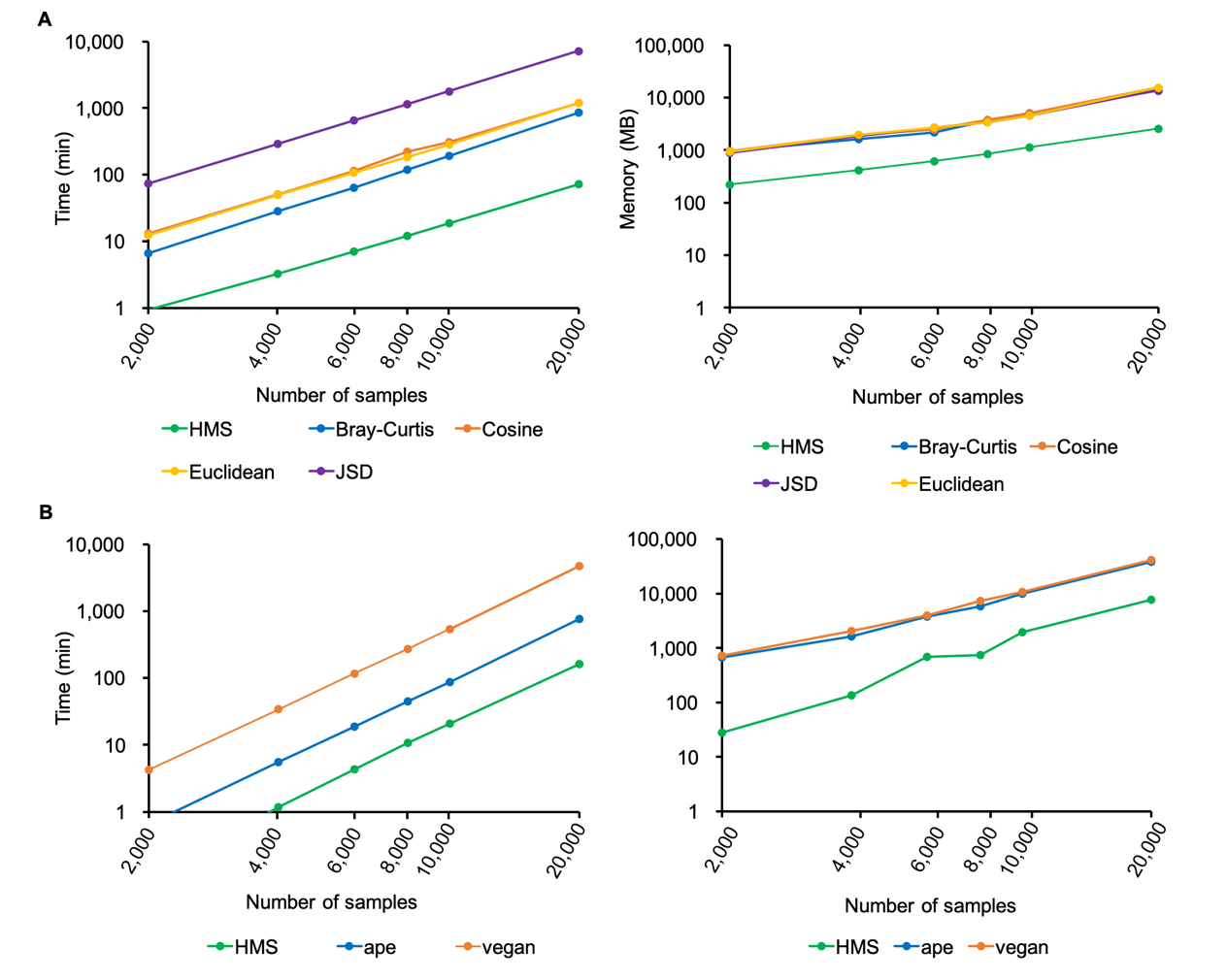


**Fig. S1. Running time and peak memory usage of (A) distance matrix calculation and (B) PCoA in log-scale.**

**Supplementary tables**

**Table S1. Performance comparison between HMS and benchmark methods in distance matrix computing.**

**(A) Total running time (minutes).**

| **# of samples** | **2,000** | **4,000** | **6,000** | **8,000** | **10,000** | **20,000** |
| --- | --- | --- | --- | --- | --- | --- |
| HMS | 0.93 | 3.27 | 6.95 | 12.02 | 18.62 | 72.55 |
| HMS with single core | 80.60 | 325.28 | 772.75 | 1,249.60 | 1,979.83 | 7,976.53 |
| Bray-Curtis | 6.60 | 27.97 | 63.33 | 119.47 | 189.98 | 857.37 |
| Cosine | 12.96 | 50.40 | 114.41 | 219.90 | 310.52 | 1,184.71 |
| Euclidean | 12.38 | 49.34 | 105.87 | 184.97 | 283.19 | 1,190.35 |
| JSD | 73.25 | 291.47 | 647.60 | 1,131.00 | 1,809.92 | 7,289.09 |
| Mean of benchmark methods | 26.30 | 104.79 | 232.80 | 413.84 | 648.40 | 2,630.38 |

**(B) Peak RAM (MB) usage.**

| **# of samples** | **2,000** | **4,000** | **6,000** | **8,000** | **10,000** | **20,000** |
| --- | --- | --- | --- | --- | --- | --- |
| HMS | 222 | 414 | 621 | 849 | 1,126 | 2,560 |
| Bray-Curtis | 951 | 1,638 | 2,150 | 3,686 | 5,120 | 13,619 |
| Cosine | 958 | 1,945 | 2,457 | 3,788 | 5,017 | 15,872 |
| Euclidean | 932 | 1,952 | 2,668 | 3,385 | 4,512 | 15,878 |
| JSD | 894 | 1,843 | 2,662 | 3,379 | 4,505 | 14,029 |
| Mean of benchmark methods | 934 | 1,844 | 2,484 | 3,560 | 4,788 | 14,849 |

**Table S2. Performance comparison between HMS and benchmark methods in PCoA.**

**(A) Total running time (minutes).**

| **# of samples** | **2,000** | **4,000** | **6,000** | **8,000** | **10,000** | **20,000** |
| --- | --- | --- | --- | --- | --- | --- |
| HMS | 0.10 | 1.17 | 4.28 | 10.72 | 20.65 | 160.72 |
| HMS with single core | 0.38 | 3.50 | 10.52 | 24.52 | 47.38 | 373.58 |
| ape | 0.65 | 5.53 | 18.65 | 44.72 | 86.18 | 755.97 |
| vegan | 4.22 | 33.75 | 115.75 | 271.63 | 536.27 | 4,728.93 |
| Mean of benchmark methods | 2.43 | 19.64 | 67.20 | 158.18 | 311.23 | 2,742.45 |

**(B) Peak RAM (MB) usage.**

| **# of samples** | **2,000** | **4,000** | **6,000** | **8,000** | **10,000** | **20,000** |
| --- | --- | --- | --- | --- | --- | --- |
| HMS | 28 | 136 | 695 | 735 | 1,945 | 7,680 |
| ape | 674 | 1,638 | 3,788 | 5,734 | 9,932 | 37,683 |
| vegan | 723 | 2,048 | 3,993 | 7,270 | 10,752 | 40,858 |
| Mean of benchmark methods | 699 | 1,843 | 3,891 | 6,502 | 10,342 | 39,270 |
